# Alternative bacteriophage life cycles: the carrier state of *Campylobacter jejuni*

**DOI:** 10.1098/rsob.130200

**Published:** 2014-03-26

**Authors:** Patcharin Siringan, Phillippa L. Connerton, Nicola J. Cummings, Ian F. Connerton

**Affiliations:** Division of Food Sciences, School of Biosciences, University of Nottingham, Sutton Bonington Campus, Loughborough, Leicestershire LE12 5RD, UK

**Keywords:** *Campylobacter*, bacteriophages, carrier state life cycle, phage therapy, *Campylobacter jejuni*

## Abstract

Members of the genus *Campylobacter* are frequently responsible for human enteric disease, often through consumption of contaminated poultry products. Bacteriophages are viruses that have the potential to control pathogenic bacteria, but understanding their complex life cycles is key to their successful exploitation. Treatment of *Campylobacter jejuni* biofilms with bacteriophages led to the discovery that phages had established a relationship with their hosts typical of the carrier state life cycle (CSLC), where bacteria and bacteriophages remain associated in equilibrium. Significant phenotypic changes include improved aerotolerance under nutrient-limited conditions that would confer an advantage to survive in extra-intestinal environments, but a lack in motility eliminated their ability to colonize chickens. Under these circumstances, phages can remain associated with a compatible host and continue to produce free virions to prospect for new hosts. Moreover, we demonstrate that CSLC host bacteria can act as expendable vehicles for the delivery of bacteriophages to new host bacteria within pre-colonized chickens. The CSLC represents an important phase in the ecology of *Campylobacter* bacteriophage.

## 2. Introduction

Human enteric disease caused by members of the genus *Campylobacter* is widespread throughout the world. Acquisition of infection can arise from food- and water-borne sources, but notably often occurs through the consumption of contaminated poultry products [1]. Healthy poultry frequently harbour campylobacters in large numbers, as a part of their normal intestinal flora, which are subsequently transferred to poultry meat during processing. Various strategies to reduce contamination have been suggested and evaluated [1]. Among these is the novel approach of using *Campylobacter*-specific bacteriophages, which are natural predators of the pathogen, but this requires an understanding of their complex life cycles to enable successful exploitation [2]. Bacteriophage life cycles are generally ascribed as being either lytic or lysogenic [3]. During the lytic cycle, bacteriophage infection redirects host metabolism towards the replication of the phage nucleic acid and assembly of new phage particles, which are then released upon cell lysis. In the lysogenic life cycle, injected phage nucleic acid either integrates into the host genome or remains as a stable episome, replicating along with the host. In either case, the phage can recommence the lytic cycle either spontaneously or in response to stress. However, several alternatives to the main strategies of lysis or lysogeny have been reported [4]. A common feature among these alternate life cycles is their inherent instability, making their study problematic. Terminology related to these variant life cycles is often confused, but the two most documented types are pseudolysogeny (false lysogeny) and the carrier state [3,5].

The term pseudolysogeny has been used to define various different unstable bacteriophage–host interactions, including intersection with the carrier state [4]. A specific definition was applied by Ripp & Miller [6], where pseudolysogeny was defined as a phage–host cell interaction in which the nucleic acid of the infecting phage neither establishes a long-term, stable relationship (lysogeny) nor elicits a lytic response, but simply resides within the cell in a non-active state. This type of pseudolysogeny is usually associated with starvation conditions. As nutrient supplies are replenished, the viral genome can either establish true lysogeny or become activated to produce and release viable bacteriophage following cell lysis [7,8]. A different definition of pseudolysogeny has been used to describe a situation where the phage genome is carried as an unstable episome in the host, which is both distinct from lysogeny and can be easily cured through subculture [9,10].

The carrier state life cycle (CSLC) describes mixtures of bacteria and of bacteriophages which are in a more or less stable equilibrium [5]. A proportion of bacteria are resistant; however, the presence of some sensitive variants appears to sustain the phage population so that both thrive. A characteristic feature ascribed to the state described as CSLC is that bacteriophages are constantly being generated within the culture at the expense of a sensitive cell population. When first isolated, strains exhibiting CSLC resemble lysogens in that they appear to be resistant to superinfection and exhibit infrequent plaque formation in soft agar lawns, but differ from the majority of lysogens in that the bacteriophage nucleic acid does not appear to be integrated into the host genome. The CSLC has been observed with strictly lytic bacteriophage, for which there are several examples of such relationships for phages infecting various bacterial genera [11–18]. Moreover, the persistence of T4 bacteriophage in starved *Escherichia coli* has recently been reported to lead to the production of distinguishable phage subpopulations [19]. Bacteriophage life cycle variations need careful consideration as they are on occasion confused in their interpretation between pseudolysogeny and the carrier state [4].

Biofilms provide a protected environment in which bacteria can survive harsh conditions and/or nutrient limitation. Biofilms are composed of populations of either mixed or single bacterial species embedded in a polymeric matrix of extracellular polysaccharide, protein, lipid and nucleic acid [20,21]. We have previously examined the effect of bacteriophage treatments of *Campylobacter* biofilms using two lytic group III bacteriophages, CP8 and CP30A, and demonstrated that these bacteriophages can reduce the numbers of viable bacteria and disperse the matrix [22]. However, we noted profound strain-dependent differences in rates of resistance of bacteria surviving bacteriophage treatment, which prompted us to further examine the survivors of bacteriophage infection of biofilms. A large number of these isolates were found to be typical of the CSLC described above, and to spontaneously produce bacteriophages. It is postulated that the establishment of CSLC may grant a survival advantage to both bacteria and bacteriophages within biofilms. This study therefore aims to investigate the formation and physiology of CSLC *Campylobacter jejuni* and the characteristics of bacteriophages arising from the association.

## 3. Material and methods

### 3.1. *Campylobacter* strains and bacteriophages

*Campylobacter jejuni* strains PT14, PT1 (phage typing standards [23]), HPC5, TIVC9, GH2F7, BIIIC3, HPIF9, F2E3, F2C10 (broiler house isolates [24–26]), NCTC 11168 and *C. coli* PT44 (phage typing standard [23]) were routinely grown on 5% horse blood agar (BA; CM0271; Oxoid, UK; with horse blood from TCS, UK) at 42°C under microaerobic conditions for 18 h as previously described [24]. A spontaneous streptomycin-resistant (Smr) mutant of PT14 was isolated as described by Miller *et al.* [27]. *Campylobacter* cultures were resuspended in Mueller-Hinton (MH) broth (CM0337; Oxoid) at *A*_600_ 0.3–0.4 using a sterile swab and then incubated (42°C under microaerobic conditions for 18 h) to use as inoculums for biofilm formation. The microaerobic atmosphere was achieved using CampyGen (Oxoid) gas packs placed in sealed incubation boxes or using anaerobic jars employing gas replacement (85% N_2_, 5% O_2_ and 10% H_2_).

Group III bacteriophages CP8 and CP30A were isolated in the UK from poultry excreta [24]. The group II bacteriophage CP220 [28,29] was isolated from poultry meat in the UK. These phages were propagated on the bacterial hosts embedded in soft agar overlay using NZCYM (Fisher Scientific, UK) agar as previously described [24,30]. The genome sequences of bacteriophages CP8 and CP30A appear in GenBank under the respective accession numbers of KF148616 and JX569801.

### 3.2. Enumeration of campylobacters and bacteriophages

Campylobacters were enumerated as previously described [24]. Briefly, serial 10-fold dilutions were made in MRD (CM0733; Oxoid) and enumerated in triplicate on mCCDA (CM0739; Oxoid) agar with additional agar (L13; Oxoid) to a total of 2% (w/v) added to reduce swarming. Plates were incubated under microaerobic conditions at 42°C for 48 h before typical *Campylobacter* colonies were counted. Bacteriophages were enumerated using the soft agar overlay method as previously described [30]. Briefly, serial 10-fold dilutions of phage suspensions were applied as 10 µl droplets in triplicate to the surface of prepared host bacterial lawns and allowed to dry. Plates were then incubated under microaerobic conditions at 42°C for 48 h before the plaques were counted.

### 3.3. Formation and infection of biofilms

*Campylobacter* biofilms were formed as previously described [22] by incubating *Campylobacter* cultures in Petri dishes containing glass coverslips, which were then incubated at 37°C under microaerobic conditions for 120 h. After this time, 10^9^ PFU bacteriophages were applied to the biofilm and incubated at 37°C under microaerobic conditions for 24 h. The temperature and period of incubation were selected because they had previously [22] been found to provide the optimum conditions for biofilm formation, although biofilms and CSLC strains were also formed at 42°C. Subsequent experiments on the CSLC strains isolated from the biofilms were carried out at 42°C, the normal body temperature for avian species and the temperature that leads to unequivocal expression of the flagella biosynthetic genes in order to assess motility.

### 3.4. Isolation of carrier state life cycle strains from biofilms

Bacterial cells attached to the glass surfaces were detached using a pipette and resuspended in MRD (Oxoid). *Campylobacter* and bacteriophages were enumerated as described above. One hundred colonies were picked and tested for phage resistance as previously described [24]. Isolates exhibiting phage plaques within the bacterial lawns to which no external phage had been applied were selected for further analysis. Genomic DNAs were prepared, digested with *Sma*I restriction enzyme and together with undigested genomic DNA, and were subjected to pulsed-field gel electrophoresis (PFGE) as previously described [31]. Motility testing was carried out as previously described [22]. The isolates were subcultured a minimum of five times and if they continued to produce plaques from 0.2 µm filtered suspensions, the isolates were considered to be potentially CSLC.

### 3.5. Southern blotting and PCR amplification of bacteriophage DNAs

Genomic DNAs from CSLC isolates were prepared in agar blocks and separated by PFGE as previously described [31]. The PFGE gels were Southern transferred to Nylon membranes and DNAs detected using Digoxigenin-labelled probes (Roche Applied Science, Mannheim, Germany) made from either bacteriophage genomic by random prime or as specific PCR amplified DNA fragments from bacteriophage genomic DNA templates (Roche Applied Science). PCR amplification of *Campylobacter* bacteriophage DNAs generally require extension times twofold or greater than the standard synthesis capacity of the Taq-polymerase. GoTaq polymerase and nucleotide reagents (Promega, Chilworth, UK) were used to amplify phage DNAs using the discriminatory primers for bacteriophage CP8 DNA (CP853B 5′-TCGTTATACCACGGATATAG-3′ and CP854B 5′-TATAGGAGGGTTGTGAAATG-3′). These primers were designed to PCR amplify a short variable region of the bacteriophage genomes, that contains a DNA sequence insertion in CP8 (GenBank accession number KF148616) that distinguishes it from all group III bacteriophage sequences available including CP30A.

### 3.6. Transmission electron microscopy

Transmission electron microscopy (TEM) was carried out as previously described [24] using 0.5% (w/v) uranyl acetate negative stain.

### 3.7. Neutralization of CP8 with antiserum

Antiserum was raised against CsCl-density gradient-purified CP8 bacteriophage particles in rabbits (Pickcell Laboratories Ltd, The Netherlands). Bacteriophage CP8 and CP8-derived CSLC cultures were treated with either rabbit pre-immune serum or serum raised against bacteriophage CP8 (diluted 1 : 16 in PBS to affect neutralization of 10^8^ PFU CP8 within 5 min without compromising the viability of campylobacters) or chloroform (Fisher, UK; equal volume to inactivate the bacteria). Overnight growth of *Campylobacter* on blood agar plates were suspended in 10 ml PBS and pelleted by centrifugation at 13 000×*g* for 5 min. The bacteria were resuspended and washed twice in PBS before final resuspension in PBS buffer to contain approximately 8 log_10_ CFU ml^−1^. The bacteria suspension was mixed with an equal volume of diluted antiserum (final dilution 1 : 16) and incubated at 42°C for 15 min. Viable *Campylobacter* counts and bacteriophage titres were determined post-treatments as described above.

### 3.8. Efficiency of plating of the parental and carrier state life cycle derived bacteriophages

The efficiency of plating (EOP) of bacteriophages and CSLC bacteriophages was determined by enumerating bacteriophages as described above and dividing the bacteriophage titre obtained when applied to the lawns prepared from a variety of different *C. jejuni* and *Campylobacter coli* strains from laboratory stocks, by the titre obtained when applied to *C. jejuni* PT14 lawns.

### 3.9. Growth characteristics of *Campylobacter jejuni* carrier state life cycle isolates

The growth characteristics of the CSLC isolates were compared with the parental *C. jejuni* isolates as previously described [32] with slight modification. Briefly, each of the CSLC isolates and parental C. *jejuni* isolates were inoculated into 50 ml of sterile MH broth in conical flasks, to approximately 10^5^ CFU ml^−1^. The flasks were incubated with shaking at 42°C, under microaerobic conditions for 24 h. Aliquots of 100 µl were removed every 2 h for enumeration of bacteria and bacteriophages, as described above. Approximately 50 colonies were subcultured from mCCDA plates at each time point for phenotype determination. Motility was assessed as previously described [31], by inoculation of 0.4% MH agar followed by incubation for 24 h under microaerobic conditions. Motility was assessed as a function of the radius of the motility halo, with a strain being defined as motile if the halo radius exceeded 20 mm. A carrier state phenotype was defined as one where plaques could be observed following microaerobic incubation of a soft agar overlay containing the test isolate (§3.1). A phage-resistant phenotype was defined as one where 10^4^ PFU of the test phage failed to produce any plaques when applied as 10 µl droplets in triplicate.

### 3.10. Survival of *Campylobacter jejuni* and carrier state life cycle isolates under stress conditions

Cultures of C. *jejuni* PT14, HPC5 and the CSLC isolates were prepared in 50 ml of sterile MRD to contain approximately 10^5^ CFU ml^−1^ and incubation was carried out at 42°C in normal atmospheric oxygen with shaking. The sampling frequency was optimized according to viability. Campylobacters and bacteriophages were enumerated as described above. Change in viability was calculated as the percentage of the original culture that remained at each time point.

### 3.11. Adsorption of parent and carrier state life cycle bacteriophages to campylobacters

Suspensions of *C. jejuni* PT14 and HPC5 that contained approximately 10^9^ CFU ml^−1^ were prepared in MRD. The actual viable count was determined following serial dilution and incubation as described above. The parent and CSCL bacteriophages (enumerated as described above) were diluted and added to the suspensions to give a final count of 10^5^ PFU ml^−1^, then mixed and incubated, with shaking, at 42°C under microaerobic conditions and sampled every 5 min over 30 min. Samples were immediately centrifuged at 13 000×*g* for 5 min and the supernatants removed. The titre of the free bacteriophages in the supernatants was determined and used to calculate numbers of bound bacteriophages. Bacteriophage adsorption constants [33] were determined using the formula *k* = −ln (*P_t_*/*P*_0_)/*Nt*, where *P_t_* = phage titre at the time *t* (PFU ml^−1^), *P*_0_ = initial phage titre (PFU ml^−1^), *N* = bacterial density (CFU ml^−1^) and *t* = time (min).

### 3.12. Colonization of chickens with carrier state life cycle campylobacters

Protocols for the infection and treatment of birds together with measures to ensure biosecurity were as previously described [24]. For each of the four CSCL isolates and two parental controls, seven 22-day-old birds were inoculated orally with 10^7^ bacteria (CFU). The caecal contents were processed after 24 h for *Campylobacter* and bacteriophage enumeration as previously described [24].

### 3.13. Application of carrier state life cycle as a delivery mechanism of bacteriophages to chickens pre-colonized with non-carrier state life cycle *Campylobacter jejuni*

Five groups of 22-day-old chickens (*n* = 7 per group) were colonized as above with C. *jejuni* PT14Smr (streptomycin resistant). After 48 h the groups were treated as follows: Group 1, placebo (1 ml PBS); Group 2, CP8 phage (1 ml of 7 log_10_ PFU ml^−1^ in PBS); Group 3, *C. jejuni* PT14CP8CS (1 ml of 8 log_10_ CFU ml^−1^/7 log_10_ PFU ml^−1^ in PBS); Group 4, CP30 phage (1 ml of 7 log_10_ PFU ml^−1^ in PBS); and Group 5, *C. jejuni* PT14CP30ACS (1 ml of 8 log_10_ CFU ml^−1^/7 log_10_ PFU ml^−1^ in PBS). The birds were sacrificed after a further 72 h and the caecal contents collected. Phages and *Campylobacter* were enumerated as previously described [24]. *Campylobacter* colonies from the enumeration plates for each bird were replicated on to streptomycin containing medium (mCCDA containing 100 µg ml^−1^ streptomycin) to confirm that the colonies enumerated corresponded to the colonization culture (streptomycin resistant) and had not arisen from the treatment cultures (streptomycin sensitive).

## 4. Results

### 4.1. Recovery of stable phage producing *Campylobacter jejuni* from phage-treated biofilms

Single colony isolates of *C. jejuni* PT 14 and HPC5-recovered post-bacteriophage treatment of static biofilms were unexpectedly observed to produce background plaques when propagated in soft agar overlays. Plaque formation was observed in 10% of the *C. jejuni* PT14 recovered, and between 40 and 90% of *C. jejuni* HPC5. All were sensitive to superinfection by other phages. The ability of these isolates to form plaques could not be removed by repeated washing, centrifugation and resuspension of the cells, and was stably maintained through five successive subcultures from single colonies on BA plates. These features typify the phage CSLC where bacteria and bacteriophages remain associated in equilibrium. Stable CSLC cultures obtained from the phage treatment of biofilms were designated as *C. jejuni* PT14CP8CS, PT14CP30ACS, HPC5CP8CS and HPC5CP30ACS.

### 4.2. Carrier state life cycle phages correspond with the treatment phages

In order to identify whether the phages produced by the CSLC cultures correlated with those used for the initial treatment, PCR amplification of CP8 and CP30A phage DNAs with phage-specific primers (CP853B and CP854B) produced fragment sizes of 282 and 190 bp, respectively, whereas *Campylobacter* genomic DNAs failed to amplify these fragments. These data were further confirmed by DNA sequencing of PCR amplified fragments from CSLC bacteriophage and total culture DNAs. It is clear that these bacteriophages retain the genotypes of the parent phage from all host sources and are not the consequence of excision of an unrelated prophage or contamination with alternative bacteriophages.

**Figure 1.**
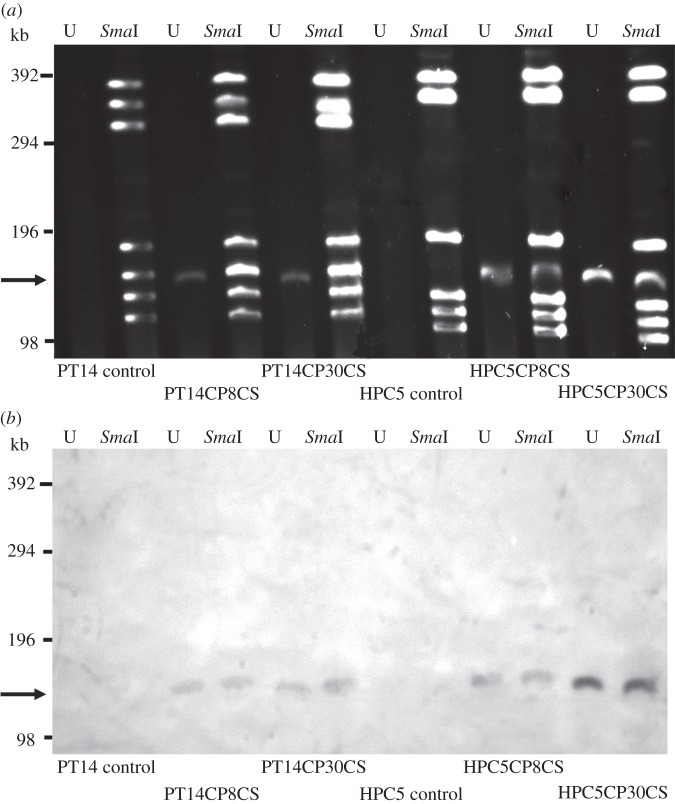
PFGE and Southern blot of genomic DNA from *C. jejuni* CSLC cultures. PFGE and Southern blot analysis was carried out to establish that the associated phage genomes were not integrated into the host chromosome. (*a*) PFGE of *C. jejuni* PT14 and HPC5 carrier strains recovered from biofilms treated with either CP8 or CP30A bacteriophages together with controls not treated with bacteriophages. U, undigested genomic DNA (bacterial genomic DNA remained in the well under these electrophoretic conditions); *Sma*I, genomic DNA digested with *Sma*I restriction enzyme. DNA bands of approximately 140 kb (indicated by arrow) occur in the undigested preparation and remain within the *Sma*I digested DNA fragments; these were identified in the DNA preparations of *C. jejuni* CSLC strains but not their non-phage-treated progenitors. (*b*) Southern blot of PFGE above. The Southern blot was hybridized with a CP8 DNA probe, which cross-hybridizes with CP30A DNA, and clearly shows the 140 kb fragments to be phage DNA. Size markers indicate the positions of concatenated lambda DNAs loaded on the PFGE.

To rule out changes in the *Campylobacter* genome due to potential integration of phage DNA, *Sma*I restriction digestion and separation of large DNA fragments by PFGE was carried out (figure 1*a*). The distinctive patterns of the *Sma*I fragments for each of the *Campylobacter* isolates were maintained. However, additional discrete bands of approximately 140 kb corresponding to the expected size of the phage genome [24,34] were identified in the CSLC isolates but not their untreated progenitors (figure 1*b*). *Sma*I digestion of the *C. jejuni* PT14 chromosome yielded seven fragments, one of which was approximately the same size as the phage genome (140 kb) and consequently produced an intense double band on the PFGE gel that is clearly visible in the undigested samples. A Southern transfer of the PFGE separated total DNAs including the phage genomic DNAs was hybridized consecutively with labelled CP8 and CP30A bacteriophage DNA probes (figure 1*b*). These results confirmed that the DNA band of 140 kb in the CSLC isolates corresponds to the genetic material of the treatment phages, and that the phage genomic DNAs are not integrated into the bacterial chromosome in the CSLC isolates recovered from biofilms.

The continued presence of bacteriophages appearing as propagatable clear plaques in bacterial lawns of single colony subcultures coupled with evidence for the presence of bacteriophage DNA in these cultures support the idea that the bacteriophage virulent life cycle had been interrupted, and that phages are intimately associated with the host bacteria either as pre-absorbed phage particles or represented as internalized DNAs. These observations are consistent with the criteria for CSLC cultures [4].

### 4.3. Carrier state life cycle isolates are non-motile

**Figure 2.**
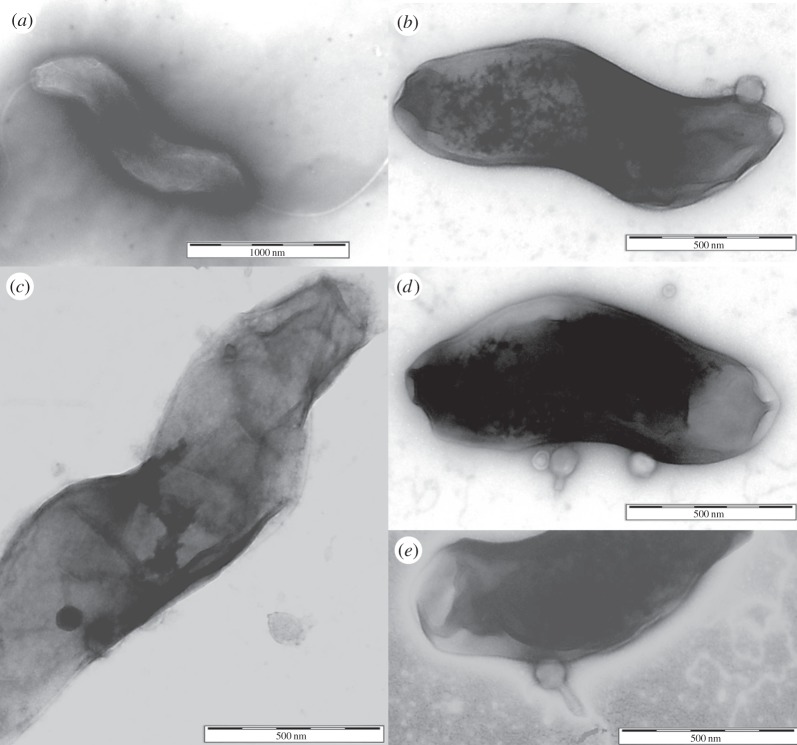
TEM images of CSLC stained with uranyl acetate. (*a*) Typical *C. jejuni* HPC5 bacterium showing intact flagella at each end. (*b–e*) CSLC cultures from BA plates showing bacteriophage particles in association with the surface of *Campylobacter* cells but with no evidence of infection structures or host lysis. It is also notable that the bacteria have no flagella.

Microscopic examination of the CSLC cultures by light microscopy revealed that the campylobacters were non-motile and this was confirmed by their inability to swarm on soft agar plates. TEM images of CSLC cultures showed bacteriophage particles in association with the surface of *Campylobacter* cells but with no evidence of infection structures or host lysis. It was also notable that the bacteria had no flagella (figure 2).

### 4.4. Treatment of carrier state life cycle isolates with phage neutralizing antiserum

To assess whether the bacteriophages remain adhered to the bacteria but accessible to the environment, CSLC cultures washed by repeated centrifugation were treated with rabbit anti-CP8 phage serum. Table 1 shows that the serum treatment neutralized free phages in the absence of bacteria and also neutralized phages that were simply mixed with bacteria. However, the anti-CP8 serum did not neutralize the phages that were associated with the CSLC bacteria, suggesting that the phages were protected either as intimately surface-bound particles that remain quiescent until sensitive host bacteria are available or are internalized as preformed phages or phage DNA. Chloroform treatment was shown to inactivate the bacteria but not the phages. CSLC phages could be recovered post-chloroform treatment suggesting that pre-assembled phage particles are associated with the bacteria, but chloroform treatment reduced the phage titre significantly by approximately 1 log_10_ PFU (*p* < 0.05), also suggesting a contributory dependence on host replication.

**Table 1.** Treatment of *C. jejuni*, their carrier state bacteria and CP8 phage with either pre-immune or anti-phage sera or chloroform.

combination	pre-immune serum^a^		anti-phage serum^a^		chloroform^a^	
	bacteria count log_10_ CFU ml^−1^	phage count log_10_ PFU ml^−1^	bacteria count log_10_ CFU ml^−1^	phage count log_10_ PFU ml^−1^	bacteria count log_10_ CFU ml^−1^	phage count log_10_ PFU ml^−1^
CP8 phage only	0	5.3 (±0.5)	0	0	0	5.8 (±0.4)
HPC5 parent bacteria only	7.8 (±0.4)	0	7.8 (±0.6)	0	0	0
HPC5 parent bacteria mixed with phage	7.3 (±0.6)	4.8 (±0.4)	6.9 (±0.7)	0	0	5.2 (±0.6)
HPC5CP8CS	7.6 (±0.6)	7.5 (±0.5)	7.1 (±0.3)	7.6 (±0.4)	0	6.1 (±0.9)
PT14 parent bacteria only	7.2 (±0.4)	0	7.0 (±0.5)	0	0	0
PT14 parent bacteria mixed with phage	6.8 (±0.8)	4.9 (±0.6)	6.5 (±0.5)	2.0 (±0.8)	0	5.1 (±0.6)
PT14CP8CS	7.1 (±0.3)	6.9 (±0.6)	7.0 (±0.6)	6.8 (±0.6)	0	5.2 (±0.4)

^a^Values are recorded ±s.d. in parentheses (*n* = 3); zero values are below the limit of detection of 2 log_10_ CFU or PFU ml^−1^.

### 4.5. Comparison of carrier state life cycle phages with parental phages

The ability of the phages CP8 and CP30A and the CSLC phages were compared with respect to their ability to bind and replicate on different *C. jejuni* strains at 42°C under microaerobic conditions. The bacteriophage adsorption constants (*k*) from these combinations are presented in table 2, where in general the CSLC phages show similar *k*-values to the parental phages. However, the *k*-values for all the phages adsorbing to *C. jejuni* 11168 are consistently lower than those obtained for the other two strains of C. *jejuni* tested. Host range and replication were assessed by the EOP with respect to plaque formation on *C. jejuni* PT14 that was capable of replicating all phages (table 3). The CSLC phages were unaltered with respect to the EOPs recorded for the strains from which they had been selected but exhibited some significant changes with respect to other host *Campylobacter* strains. Most notably, the CP30A CSLC derivatives show greater than 10-fold changes in EOP compared with phage produced by conventional lysis (indicated in italics in table 3).

**Table 2.** Adsorption constants comparing CSLC and parent phages of CP8 and CP30A.

	adsorption constant (*k*) × 10^−10^ ml min^−1a^		
adsorption strain tested	*C. jejuni* PT14	*C. jejuni* HPC5	*C. jejuni* 11168
CP8 parent phage propagated on PT14	1.1 (±0.15)	1.35 (±0.10)	0.34 (±0.15)
CP8 CSLC phage from PT14CP8CS	1.35 (±0.35)	1.31 (±0.26)	0.33 (±0.12)
CP8 parent phage propagated on HPC5	2.09 (±0.20)	1.01 (±0.27)	0.89 (±0.13)
CP8 CSLC phage from HPC5CP8CS	1.22 (±0.26)	1.32 (±0.28)	0.59 (±0.12)
CP30A parent phage propagated on PT14	1.13 (±0.16)	1.53 (±0.35)	0.81 (±0.33)
CP30A CSLC phage from PT14CP8CS	1.33 (±0.25)	1.61 (±0.44)	0.62 (±0.25)
CP30A parent phage propagated on HPC5	1.74 (±0.26)	2.05 (±0.71)	0.68 (±0.22)
CP30A CSLC phage from HPC5CP8CS	1.25 (±0.30)	1.43 (±0.29)	0.97 (±0.41)

^a^Values are recorded ±s.d. in parentheses (*n* = 3).

**Table 3.** EOP of the parental and CSLC-derived phages compared to *C. jejuni* PT14.

	EOP of CP8 derivatives^b^			EOP of CP30A derivatives^b^		
strain	parent CP8^a^	phage from HPC5CP8CS	phage from PT14CP8CS	parent CP30A^a^	phage from HPC5CP30ACS	phage from PT14CP30ACS
*C. jejuni* PT14	1	1	1	1	1	1
*C. jejuni* PT1	0.000006 (±0.000003)	0.000004 (±0.000002)	0.00001 (±0.00004)	0.000005 (±0.000002)	0.000002 (±0.000004)	*0.0003* (±*0.0001*)
*C. coli* PT44	0.04 (±0.02)	0.10 (±0.05)	0.016 (±0.012)	0	0	0
*C. jejuni* TIVC9	0	0	0	0.0006 (±0.0003)	*0.02* (±*0.016*)	0.0001 (±0.0002)
*C. jejuni* HPC5	0.85 (±0.35)	0.32 (±0.18)	0.40 (±0.12)	0.76 (±0.33)	1 (±0.78)	0.40 (±0.16)
*C. jejuni* GH2F7	0	0	0	0.00003 (±0.00004)	*0.0014* (±*0.0058*)	*0*
*C. jejuni* BIIIC3	0	0	0	0.36 (±0.33)	0.44 (±0.32)	*0.03* (±*0.04*)
*C. jejuni* HPIF9	1.33 (±0.48)	*0.1* (**±***0.2*)	*0.012* (±*0.008*)	0.57 (±0.31)	1 (±0.54)	*0.02* (±*0.04*)
*C. jejuni* F2E3	0	0	0	0.45 (±0.32)	0.28 (±0.40)	0.30 (±0.39)
*C. jejuni* F2C10	0	0	0	0.10 (±0.13)	*0*	*0.000005* (±*0.000004*)
*C. jejuni* 11168	0.24 (±0.22)	0.40 (±0.17)	0.12 (±0.07)	0.024 (±0.020)	*0.72* (±*0.34*)	0.006 (±0.005)

^a^Phages CP8 and CP30A were propagated by lysis of *C. jejuni* PT14.

^b^Values are recorded ±s.d. indicated in parentheses (*n* = 3), and values with a greater than 10-fold change in EOP compared with the parental phages are indicated in italics.

### 4.6. Comparison of general growth characteristics of the carrier state life cycle bacteria with the parental bacteria

**Figure 3.**
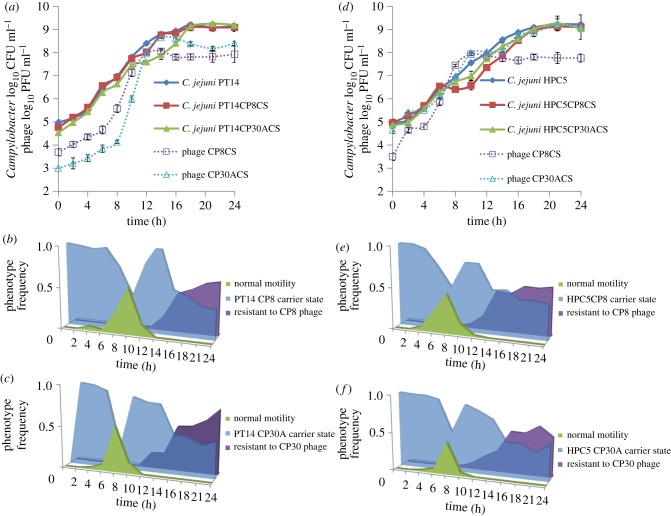
Growth curves of CSLC isolates and phages incubated under microaerobic conditions with their corresponding motility, phage resistance and carrier state phenotype frequencies. Growth curves were performed to investigate how the CSLC campylobacters behave in the presence of persistent phage infection compared with their parental strains. The phenotypic frequencies for each time point are indicated below the corresponding growth curves, where single colony isolates were scored independently for normal motility, phage resistance and carrier state. (*a*) Growth curves of *C. jejuni* PT14, PT14CP8CS, PT14CP30ACS with phage titres of CP8CS and CP30ACS; (*b*) frequencies of PT14CP8CS culture phenotypes; (*c*) frequencies of PT14CP30ACS culture phenotypes; (*d*) growth curves of *C. jejuni* HPC5, HPC5CP8CS, HPC5CP30ACS with phage titres of CP8CS and CP30ACS; (*e*) frequencies of HPC5CP8CS culture phenotypes; (*f*) frequencies of HPC5CP30ACS culture phenotypes. For a full definition of the phenotypes scored, see §3.9.

The growth characteristics of the CSLC bacterial strains were compared with those of the parental bacterial strains to detect possible changes in phenotype that might explain why the population of CSLC bacteria was not fully lysed by phage despite retaining sensitivity to them (figure 3). The early exponential phase generation times (*g*) and growth rate constants (*µ*) are shown in table 4. The doubling time and growth rates of the *Campylobacter* controls and all of the CSLC were not significantly different. However, the CSLC growth curves show a marked reduction in growth between 12 and 14 h for the *C. jejuni* PT14 CSLC derivatives (figure 3*a*) and between 8 and 10 h for the *C. jejuni* HPC5 CSLC derivatives (figure 3*d*) compared with the parental strains. The bacterial growth reductions are accompanied by substantial rises in the phage titre, notably when the viable count reached 5 log_10_ CFU ml^−1^ for the PT14 derivatives and 7 log_10_ CFU ml^−1^ for the HPC5 derivatives. The numbers of bacteriophages reached a maximum at 12 h post-incubation for both PT14 and HPC5 CSLC cultures.

**Table 4.** Generation times and growth rate constants of *C. jejuni* PT14, HPC5 and CSLC cultures under microaerobic conditions in early exponential phase. The data represent early exponential phase values before the rise in phage titre evident in figure 3. Values are recorded ± s.d. in parentheses (*n* = 5).

strain	generation time, *g* (h)	growth rate constant, *µ* (h^−1^)
*C. jejuni* PT14	0.916 (±0.032)	0.757 (±0.057)
*C. jejuni* PT14CP8CS	0.949 (±0.018)	0.730 (±0.049)
*C. jejuni* PT14CP30ACS	0.976 (±0.020)	0.710 (±0.029)
*C. jejuni* HPC5	0.894 (±0.037)	0.775 (±0.026)
*C. jejuni* HPC5CP8CS	0.952 (±0.047)	0.728 (±0.025)
*C. jejuni* HPC5CP30APCS	0.934 (±0.013)	0.742 (±0.021)

To investigate any phenotypic differences within the bacterial populations that could account for discontinuity of growth in the CSLC in broth cultures, discrete single colonies were subcultured from the enumeration plates of the growth curve experiments at each sample time point and then tested independently for their motility and phage sensitivity. The time-dependent changes in the frequency of phage resistance and motility are recorded in figure 3*b*,*c* for the *C. jejuni* PT14 and figure 3*e*,*f* for *C. jejuni* HPC5 CSLC derivatives. Phage-resistant colonies appeared in PT14C8CS and PT14CP30A at 12 h, which coincided with the interval at which the growth rate fell and the rise in phage titre. Similarly, phage-resistant colonies appeared in HPC5CP8CS and HPC5CP30ACS at 10 h, which also coincided with the reduction in bacterial growth and the rise in phage titre. All the phage-resistant colonies recovered were non-motile. Colonies that proved to be motile appeared at 4 h with peak frequencies at 8–10 h before the fall in growth rate and the rise in phage titre, and returned to zero at 14 h, which coincided with the resumption of the growth rate.

### 4.7. Survival of *Campylobacter jejuni* parental strains and carrier state life cycle cultures under stress conditions

**Figure 4.**
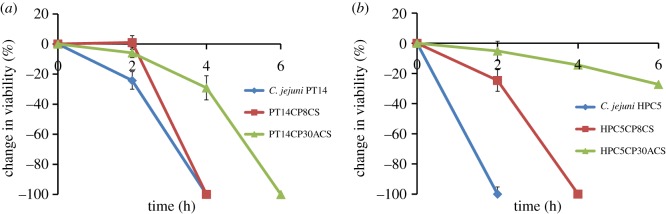
Survival of *C. jejuni* parental strains and CSLC cultures in normal atmospheric oxygen conditions. Cultures were incubated at 42°C under nutrient limitation in normal atmospheric oxygen with shaking. The sampling frequency was optimized according to viability. Change in viability was calculated as the percentage of the original culture that remained at each time point. (*a*) *Campylobacter jejuni* PT14, PT14CP8CS, PT14CP30ACS. (*b*) *Campylobacter jejuni* HPC5, HPC5CP8CS, HPC5CP30ACS.

Survival studies under oxidative stress conditions, with nutrient limitation, were performed in order to determine whether the CSLC cultures differed from their parental strains. First, the survival of the *C. jejuni* parental strains and the CSLC variants under nutrient limitation were assessed at 42°C under microaerobic conditions. The results show that all the cultures were able to survive equally well under microaerobic conditions throughout the 6 h experiment (results not shown) with the bacteriophage titres also remaining constant in the CSLC cultures. Further studies were performed by exposing the parental strains and the CSLC cultures to oxidative stress conditions by shaking flasks in normal atmospheric oxygen at 42°C. As expected for strictly microaerophilic organisms, the numbers of viable cells declined after exposure to normal atmospheric oxygen conditions at 42°C. Interestingly, the CSLC isolates decline significantly less over the first 2 h than either of the parental isolates. After 4 h of exposure, the viable count fell below the limit of detection (more than 2 log_10_ CFU ml^−1^) for both *C. jejuni* PT14 and PT14CP8CS. By contrast, PT14CP30ACS cells remained viable after this time but were no longer detectable after a total of 6 h exposure (figure 4*a*). Similarly, the HPC5 CSLC strains retained greater viability than the parent strain after 2 h of exposure to atmospheric oxygen, which had fallen below the limit detection. HPC5CP30ACS persisted beyond the 6 h duration of the experiment while HPC5CP8CS fell below the limit of detection after 4 h of exposure (figure 4*b*). The CSLC *C. jejuni* isolates could therefore survive significantly longer than the wild-type strains in atmospheric oxygen in nutrient-limited medium. The numbers of bacteriophages remained constant throughout the 6 h experiment (data not shown).

### 4.8. Carrier state life cycle cultures do not efficiently colonize chickens

The caecal colonization potentials of stable CSLC cultures recovered from biofilms were assessed in 22-day-old broiler chickens (*n* = 7). Control inoculums of *C. jejuni* PT14 and HPC5 colonized efficiently after 24 h at levels (6.1 log_10_ (±0.7) CFU g^−1^ for PT14 and log_10_ 6.4 (±0.4) CFU g^−1^ for HPC5), similar to those recorded previously [24]. However, none of the CSLC cultures colonized the birds and no bacteriophages were recovered.

### 4.9. Carrier state life cycle host bacteria can act as an expendable vehicle for the delivery of bacteriophages

While the CSLC cultures were unable to colonize chickens directly, they were however able to act as a vehicle to deliver phages to chickens that had been pre-colonized with *Campylobacter*. Chickens pre-colonized with streptomycin-resistant *C. jejuni* PT14 (48 h) were treated with 7 log_10_ PFU either as phage suspensions (CP8 or CP30) or as constituents of CSLC cultures (PT14CP8CS or PT14CP30ACS), or given a phage-free diluent control. Enumeration of the total and streptomycin-resistant *Campylobacter* populations after a further 72 h revealed significant reductions in the pre-colonizing streptomycin-resistant *C. jejuni* present in the caecal contents of the birds administered with phages (*p* < 0.05) and the absence of any streptomycin-sensitive CSLC bacteria (table 5). Reductions in the caecal contents between 1.7 and 2.3 log_10_ CFU g^−1^ were recorded compared with phage-free *Campylobacter* colonized control chickens. These results were similar to the reductions in *Campylobacter* counts reported when bacteria-free phage preparations were fed to chickens [24].

**Table 5.** Bacteriophages from CSLC isolates infect campylobacters in pre-colonized broiler chickens. Chickens were pre-colonized for 48 h with streptomycin-resistant *C. jejuni* PT14 before treatment. n.a., not applicable; n.d., not detected.

treatment	mean *Campylobacter* log_10_ CFU g^−1^ caecal contents (±s.d.)	log_10_ reductions in *Campylobacter* means compared with controls	mean phage log_10_ PFU g^−1^ caecal contents (±s.d.)
Placebo	6.9 (±0.7)	n.a.	n.d.
CP8 phage	4.6 (±0.8)	−2.3	5.6 (±0.1)
PT14CP8CS	5.2 (±0.3)	−1.7	5.4 (±0.2)
CP30 phage	5.2 (±0.8)	−1.7	5.4 (±0.3)
PT14CP30ACS	4.9 (±0.4)	−2.0	5.3 (±0.2)

## 5. Discussion

The ability of susceptible bacteria to survive attack by bacteriophages is often assumed to be associated with the selection mutations in various bacterial genes essential for phage infection, often on the basis of experiments carried out in the laboratory using rich culture media. In the natural environment, the same mutations may prove disastrous for survival in challenging conditions; consequently, specific mechanisms of phage evasion have evolved (reviewed in [35]). With this in mind, it was of interest that we found the majority of the isolates recovered following phage treatment of *Campylobacter* biofilms were not in fact classic resistant mutants but were strains that coexist with the treatment phages in a carrier state. The phage titres in these cultures remained approximately equivalent to the numbers of viable bacteria upon subculture, implying that phage replication was continuing within a subpopulation of sensitive cells, while the remaining bacteria were capable of evading phage infection despite their close proximity.

The identification of the CSLC in the *Campylobacter* isolates raised a number of questions regarding how the equilibrium of sensitive and insensitive bacteria could be maintained in the presence of a large phage population. Initial experiments examined the possibility of a lysogenic association, although as members of the *Myoviridae* with gene contents reminiscent of the prototype virulent bacteriophage T4 this was considered unlikely [36–38]. Southern blots of PFGE separated cellular DNAs demonstrated that the phage genomes remained independent of chromosomal DNA with no evidence for any form of integration. It has been suggested that the persistence of phage infection in bacterial cultures could be mediated by slow adsorption rates of the phages, allowing the host time to divide before the cellular apparatus is subverted [15]. However, this did not appear to be the case for the bacteriophages produced by *Campylobacter* CSLC strains, which appeared to possess bacteriophage adsorption constants (*k*) similar to those propagated by conventional lysis. Treatment with anti-phage serum confirmed that while a proportion of the phage population were free within the culture, the majority of the phage population (70–90%) were closely associated with their host, either bound to the outside of the cell and sheltered from antiserum treatment by surface components, for example capsular polysaccharide, or retained within the host cell with the possibility that the phage genomes are carried as episomes. Chloroform treatment of pre-washed CSLC cultures did not eliminate the bacteriophages, suggesting that pre-assembled phage particles are associated with the bacteria and that the fate of the phages is not dependent on the fate of the host. However, chloroform treatment significantly reduced the phage titre by more than 1 log_10_ PFU, suggesting that carriage of the bacteriophages is not entirely passive and that host-dependent replication contributes to the observed phage titre. Examination of the growth curves of the CSLC cultures revealed that they experienced a temporary cessation or decline in growth rate that was accompanied by a sharp rise in phage titre. Orthodox phage-infected *Campylobacter* cultures experience a synchronized population crash at comparable bacterial densities owing to phage lysis [32]. The change in growth rate in the CSLC cultures could be accounted for if a subpopulation of the bacteria were infected by phage completing a lytic life cycle against a background of bacteria that are not subject to lysis and continue to grow. Consistent with this point of view is the appearance of a motile subpopulation in all cultures prior to the reduction in growth rate, which are effectively wild-type with respect to their phage sensitivity. The concomitant rise in phage titre is likely to be due to infection and replication within the motile subpopulation, and the reason why representatives of this population were no longer recoverable on the bacterial enumeration plates upon resumption of bacterial growth with the 3–4 log_10_ increase in phage titre increasing the probability of infection. Examination of the carrier state bacteria of *Campylobacter* propagated on solid medium by TEM produced images in which phages were associated with the surface of the bacterial cells, but none were observed to be oriented with their baseplate engaged in a manner typical of infection structures. However, the electron micrographs also revealed that the flagella of these cells were truncated, accounting for the non-motile phenotype of the carrier state cultures. The presence of functional flagella is generally required for bacteriophage infection of campylobacters. The majority of phage-resistant types recovered post-infection of laboratory cultures are impaired in motility [26,31] and mutants expressing a paralysed flagella have been shown not to support bacteriophage infection [39]. The non-flagellated bacteria are therefore unlikely to support conventional phage infection and will contribute to the basis by which phages persist in the carrier state. Motility is considered a critical factor in the ability of campylobacters to colonize and cause pathogenesis [40], and accordingly CSLC isolates were unable to colonize chickens. These characteristics make them an important environmental reservoir for phage dissemination and of potential technological use as a continuous source of phages for therapeutic and biosanitization applications in the food and agriculture industries that are aimed at reducing human exposure to campylobacters [2].

**Figure 5.**
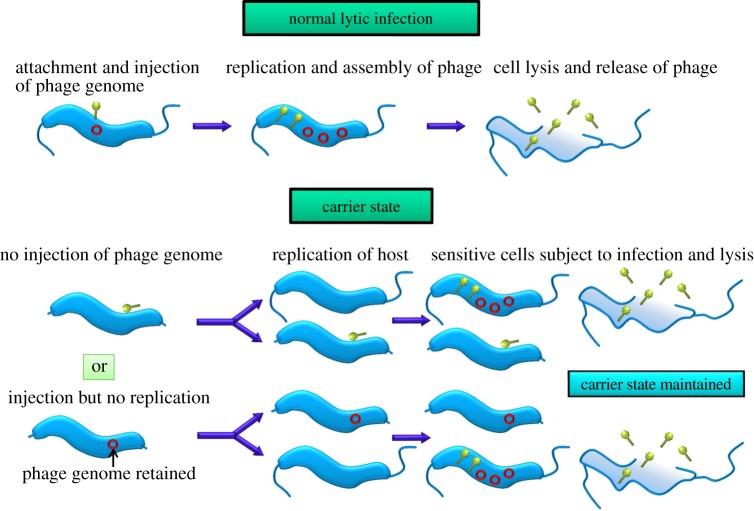
Schematic diagram of how carrier state differs from normal lytic infection in campylobacters. Models accounting for the maintenance of CSLC in *Campylobacter* envisage an equilibrium between insensitive cells that are able to survive phage attack and divide to produce sensitive host cells that support the replication of bacteriophages.

Alternative models to account for the propagation of the phages and the continued growth of the host *Campylobacter* in CSLC cultures are illustrated in figure 5. However, the outcomes of these models in terms of the formation of a subpopulation of progeny bacteria upon which the bacteriophages can replicate are similar. Our models thus envisage that equilibrium is maintained between insensitive cells that survive phage attack, which are able to divide to produce sensitive host cells that support phage replication. Evidence for the asymmetric partition of a persistent unintegrated chromosome of bacteriophage P22 in *Salmonella* Typhimurium has recently been reported, in which a phage moron gene (*pid*) was found to interact with the repressor of the host *dgo* operon, leading to derepression of genes required for d-galactonate utilization in daughter cells that receive P22 [41].

The physiology and survival characteristics of the CSLC strains were investigated under oxygen- and nutrient-limited conditions to assess whether the continued presence of the bacteriophages could affect survival in adverse conditions. No growth of any *C. jejuni* strain or their CSLC derivatives was observed under aerobic conditions, and the absence of growth did not enable phage propagation under these conditions. Maximal growth was achieved under microaerobic conditions and therefore the CSLC bacteria remain microaerophiles. However, CSLC bacteria in general show an increase in their ability to survive oxidative stresses under nutrient-limited conditions compared with their parental strains. All CSLC strains exhibit greater aerotolerance under nutrient limitation but notably the CSLC cultures formed with bacteriophage CP30A survived exposure to atmospheric oxygen for longer periods. The chicken-derived strain *C. jejuni* HPC5 exhibited the greatest tolerance with a reduction in viability of only 20% over 6 h. Examination of the differences in the gene contents between the CP8 and CP30A phage genomes did not reveal any functional characteristics that could account for the differences in aerotolerance (eight unidentified reading frames present in CP30 that are absent in CP8). However, any effects are unlikely to be direct but rather modulated through the host bacteria.

The development of aerotolerance under nutrient limitation must be considered a key attribute to enhance the survival of the bacteria upon leaving the low oxygen and relatively nutrient-rich environment of the animal intestine. As an evolutionary adaptation for the bacteriophage, the ability to maintain an association with the host in the extra-intestinal environment will ensure that a proportion of the phage population will not be irrecoverably dispersed in a habitat of low replicative probability for the host bacteria, and will benefit from the mechanisms by which the host bacteria can survive to be ingested and access the intestinal tracts of animals. The bacteriophage particles of CSLC cultures are largely preformed and remain capable of infecting new hosts despite adverse conditions, leading to slow or no growth of the bacteria carrying them. Moreover, because the phage particles are preformed and infectious, the inability of the CSLC host bacteria to efficiently colonize chickens does not prevent the phages from finding new host bacteria but rather presents the opportunity to infect and exploit the host bacteria dominating the new niche. In this context, the extra-intestinal environment has the characteristics of an ecological sink and the CSLC, a mechanism by which *Campylobacter* bacteriophages are able to survive and migrate from a non-productive habitat. It seems probable that mechanisms similar to those described here will be represented in other bacteriophage–host combinations where there is a need to survive resource-limited conditions.

In summary, we present evidence that *Campylobacter* bacteriophages can enter into a complex relationship with their host, and that this relationship will confer advantages to the host bacteria in their ability to survive in extra-intestinal environments. *Campylobacter* phages are members of the *Myoviridae* family that have the ability to stray from a virulent life cycle in order to remain associated with their host to afford them the prospect of shelter and continued replication while travelling between host-rich source environments represented by the intestinal tracts of animal hosts.

## Notes

Animal studies were conducted under the Animals Scientific Procedures Act (1986) and were approved by the University of Nottingham local ethical review committee.
